# Compressor map regression modelling based on partial least squares

**DOI:** 10.1098/rsos.172454

**Published:** 2018-08-29

**Authors:** Xu Li, Chuanlei Yang, Yinyan Wang, Hechun Wang, Xianghuan Zu, Yongrui Sun, Song Hu

**Affiliations:** College of Power and Energy Engineering, Harbin Engineering University, Harbin, People's Republic of China

**Keywords:** diesel engine, performance modelling, compressor maps, partial least squares, regression modelling

## Abstract

In this work, two kinds of partial least squares modelling methods are applied to predict a compressor map: one uses a power function polynomial as the basis function (PLSO), and the other uses a trigonometric function polynomial (PLSN). To demonstrate the potential capabilities of PLSO and PLSN for a typical interpolated prediction and an extrapolated prediction, they are compared with two other classical data-driven modelling methods, namely the look-up table and artificial neural network (ANN). PLSO and PLSN are also compared with each other. The results show that PLSO and PLSN have a better prediction performance than the look-up table and the ANN, especially for the extrapolated prediction. The computational time is also decreased sharply. Compared with PLSO, PLSN is characterized by a higher prediction accuracy and shorter computational time than PLSO. It is expected that PLSN could save computational time and also improve the accuracy of a thermodynamic model of a diesel engine.

## 1. Introduction

The turbocharger is a vital component of the modern diesel engine. It allows the engine to increase its power density through the downsizing concept [1–3] while simultaneously decreasing fuel consumption. As is well known, the compressor and the turbine are the two most important components of the turbocharger itself. Consequently, compressor models play a significant role in the simulation and modelling of diesel engines, and, thus, it is crucial to accurately describe the compressor map. However, one of the most troublesome problems in the development of a compressor model is the strong nonlinearity of its pressure characteristics curves and efficiency characteristics curves. In general, many experimental studies on the various operating and environmental conditions of a compressor are usually conducted to obtain the performance map. However, the experimental data in the full operational range are usually very limited because of the cost and restrictions of the compressor test (table 1).

**Table 1.** Nomenclature.

PLS	partial least squares
PLS-R	partial least squares regression
PLSO	partial least squares with a power function polynomial form as the basis function
PLSN	partial least squares with a trigonometric function polynomial form as the basis function
EPL	extrapolation performance at the lower part-load operating area
IPL	interpolation performance at the lower part-load operating area
EPU	extrapolation performance at the upper part-load operating area

In the published literature, there are several compressor modelling methodologies. The most popular methodology is look-up tables [4], which use the experimental compressor mass flow rate and efficiency maps. These maps are provided by compressor manufacturers in the form of lines of constant reduced speed and efficiency. The reduced speed and efficiency at each point are calculated by linearly interpolating and extrapolating the experimental maps [5]. As is well known, a compressor has strong nonlinear relationships among its reduced speed, pressure ratio, mass flow rate and efficiency. Therefore, the predictive accuracy of a compressor map using look-up tables is based on the quantity and quality of the experimental data. The prediction performance of look-up tables greatly deteriorates when the experimental data are very limited.

To improve the accuracy of the compressor's prediction, in [6], the elliptical curve fitting method was introduced to map the fitting process. In this method, the shape of the compressor map is expressed by the mathematical equation of an ellipse. According to the locations of the centre and axes of the ellipse, the elliptical curve fitting method is divided into three branches: a fixed centre without rational axes; a fixed centre with rational axes; and a flexible centre with rational axes. The method of a fixed centre without rational axes has the worst predicted accuracy, having the lowest number of subcoefficients (20 subcoefficients) of the three branches. The method of a flexible centre with rational axes has an acceptable predictive accuracy, with up to 100 subcoefficients, but this is too complicated for compressor map modelling.

Some intelligence algorithms are also applied to compressor map modelling. The well-known artificial neural network (ANN) algorithm is widely used in many areas [7] because of its ability to process nonlinearly and store massive amounts of experimental knowledge. Theoretically, an ANN can approximate any nonlinear model and develop the relationship between the input and output variables involved in a physical process without considering the underlying theories [8–10]. Hence, ANNs have become increasingly popular in recent years for predicting the performance of a compressor map. As a type of improved ANN, a feed-forward back-propagation neural network (BPNN) introduces feed-forward and back-propagation into the ANN. This means signals feed forward and there is a deviation between the experimental data and predictive value back-propagation. Consequently, BPNNs have the capability to rapidly converge the characteristics and nonlinear mapping. The network is first trained by experimental data, then the predictive results are regarded as experimental data in the second train. Although BPNNs are effective in predicting the compressor map via interpolation, their predictive accuracy is terrible in predicting the compressor map via extrapolation [11]. Moreover, it is important to note that a large number of experimental data are necessary to sufficiently train a BPNN to obtain a highly predictive accuracy and calculation stability. As a result, BPNNs are not suitable for compressor map modelling when the experimental data are very limited [12–15].

Computational fluid dynamics (CFD) simulations were applied to estimate the compressor map numerically in several studies [16–19]. This method has been proven to have a relatively high accuracy, but it is usually quite time-consuming, which makes it impossible to predict the compressor map in an available time frame. To shorten the computational time, the CFD simulation is converted into two-dimensional (2D) methodologies [20] and mean-line (one dimensional) methodologies [21] by using empirical or semi-empirical correlations. Accordingly, the predictive accuracy is inevitably sharply decreased. Another disadvantage of these methods is that they require some knowledge about the geometry of the compressor, which is not always available as this may be the manufacturer's proprietary information.

The partial least squares (PLS) regression method is a novel multiple statistical analysis method developed from the field of chemistry [22]. The PLS method has been introduced into compressor performance prediction because of the following advantages: it is strong and robust, it requires a low sample number and it has obvious physical meaning [23,24]. Even though the prediction accuracy of the PLS method is already at a high level, there is still ongoing work to improve it further. Tian *et al*. [25] combined an ANN and PLS to predict the performance of a scroll compressor, dividing the compressor performance into nonlinear and linear portions. The nonlinear portion was simulated using an ANN, while the linear one was modelled using the PLS method. The prediction results show that this method has a higher prediction accuracy than both an ANN and a normal PLS. However, this method is too complex, and it is difficult to distinguish the nonlinear and linear portions. Chu *et al*. [26] introduced a kernel function into the PLS method, referred to hereafter as the kernel partial least squares (KPLS). The KPLS improved the prediction accuracy owing to its diversity, but the prediction accuracy can greatly change when using different types of kernel functions.

As described above, completely modelling a successful engine's thermodynamic performance mainly depends on the method used, which must express well the component characteristic maps with good extrapolation and interpolation performance. In this work, we propose a new prediction model with a simple structure with a high prediction accuracy and short computational time. A new PLS prediction model (PLSN) is proposed based on the traditional PLS model by replacing the basic function of the PLS, a power function polynomial, with a trigonometric function. The results show that, when compared with a look-up table, the BPNN and the traditional PLS method (PLSO), PLSN achieves the best prediction accuracy while having the shortest computational time. Compared with other PLS models [25,26], the prediction accuracy is of the same magnitude, but the model structure of PLSN is much simpler. The remainder of this paper is organized as follows. In §2, the simulation problems of compressor characteristics maps and some relative solutions proposed in the literature are described. In §3, an application and an analysis of the novel method proposed by the authors are presented. Finally, §4 presents the conclusions.

## 2. Methodology

### 2.1. Problem description of compressor characteristics maps

One of the most troublesome problems in the development of a component-based diesel engine performance model is compressor modelling. Fortunately, a steady-state compressor map can be used in compressor modelling, because the air flow in the compressor can be assumed to be a quasi-steady flow. In general, the compressor is modelled as a lumped element in the thermodynamic performance model. The compressor performance can be represented by four characteristic parameters: reduced flow rate, isentropic efficiency, pressure ratio and reduced speed [27]. The characteristic parameters are usually represented as a flow characteristics map and an efficiency characteristics map. Once two arbitrary characteristic parameters are confirmed, the other two characteristic parameters are also confirmed through the flow characteristics map and efficiency characteristics map.

**Figure 1.**
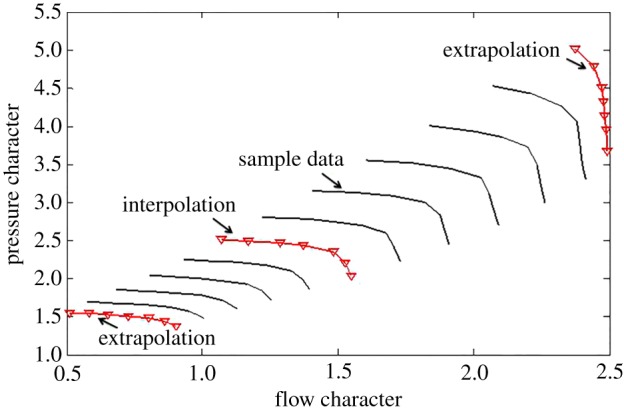
Compressor characteristics map generalization.

Usually, parts of the rotational speed characteristics curves of compressor maps, including the on-design operating point, are derived from a test bed or by means of CFD during actual thermodynamic performance modelling, and reasonable interpolation and extrapolation have to be performed in order to make good use of the flow characteristics and efficiency characteristics under a wider range of off-design characteristic areas. The experimental data shown in figure 1 are derived from the manufacturer, and the data are divided into three portions: sample data for building the predicting model, interpolation data and extrapolation data for verifying the accuracy of the prediction model.

### 2.2. Look-up table method

One of the most popular and simplest approaches is the look-up table method, in which the core algorithm is often linear or spline interpolation and extrapolation; this method has been widely used in almost every commercial thermodynamic calculation software, i.e. Krawal-modular, GT-ise, IPSEpro and Thermoflex. Owing to the sparsity of the compressor characteristics map data from the test bed or from CFD and discretization, which will reduce the amount of characteristic data further, a component characteristics table inserted into the thermodynamic performance model may cause information loss and cannot accurately represent the shape of the characteristics map. On the one hand, because of the different pressure ratio ranges for different constant rotational speed curves, the dimensional range of the pressure ratio in the table is different, which makes the interpretation of the table difficult. On the other hand, for a high, constant rotational speed curve, the flow characteristics curve is approximately vertical in the range of a comparatively small pressure ratio; however, if the data for discretization are incomplete, it is difficult to determine infinitesimal changes in the flow rate with the pressure ratio. Therefore, owing to the discretization process, a difference between the characteristics map in a table and the actual characteristics is simply unavoidable, so the sample data of compressor maps in the table should be dense and regular.

### 2.3. Artificial neural network method

**Figure 2.**
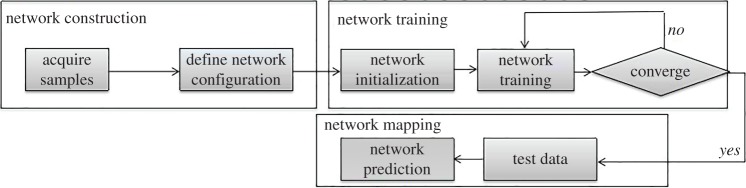
Neural network-based compressor performance modelling.

The neural network method, which can approximate any nonlinear function by selecting the appropriate network, has become an effective solution for expressing compressor characteristics maps due to its highly nonlinear mapping capacity. ANN-based compressor performance modelling involves the following three steps: network construction, network training and network mapping. A detailed schematic diagram of the process is shown in figure 2.

A BPNN [28], which is a feed-forward BPNN and one of the foremost types in the ANN family, is used as a comparative algorithm. Here, the version of the BPNN that we use is from [29].

### 2.4. A new regression analysis method: partial least squares regression

The PLS regression (PLS-R) method is a novel multiple statistical analysis method developed from the field of chemistry and proposed by S. Wold and C. Albno in 1983. PLS-R combines the basic functions of a multiple linear regression analysis, a canonical correlation analysis and a principal component analysis, and integrates the data analysis method of modelling the prediction type with a non-model-based data analysis method organically [30]. In general, a PLS-R consists of the following steps.

Suppose the number of dependent variables is *q* and the number of independent variables is *p*. To study the statistical relationship between the dependent variables and independent variables, *n* number of sample points are observed to constitute the data sheet of the dependent variables and independent variables, i.e. *X* = [*x*_1_, *x*_2_, … , *x_p_*]*_n_*_×_*_p_* and *Y* = [*y*_1_, *y*_2_, … , *y_q_*]*_n_*_×_*_q_*. Components *t*_1_ and *u*_1_ are extracted from *X* and *Y*, respectively (that is, *t*_1_ is the linear combination of *x*_1_, *x*_2_, … , *x_p_* and *u*_1_ is the linear combination of *y*_1_, *y*_2_, … , *y_q_*). During the extraction of these two components, two requirements should be noted as follows in order to accommodate the need for regression:

(i) *t*_1_ and *u*_1_ should carry the most mutation information about their own data sheets. This means the variances in *t*_1_ and *u*_1_ reach their respective maximum values.

(ii) The absolute value of the degree of the correlation of *t*_1_ and *u*_1_ achieves its maximum value.

These two requirements show that *t*_1_ and *u*_1_ should be as sufficient as possible to represent their own data sheet, while the component *t*_1_ of *X* has the strongest explanatory power for component *u*_1_of *Y*. After extracting the first components *t*_1_ and *u*_1_, PLS-R conducts the regression of *X* for *t*_1_ and the regression of *Y* for *u*_1_. If the regression equation has reached a satisfactory accuracy, the calculation terminates; if not, the second round of component extraction is conducted, making use of the residual information of *X* being interpreted by *t*_1_ and the residual information of *Y* being interpreted by *u*_1_, and so on until a satisfactory accuracy is reached. If *m* number of components *t*_1_, *t*_2_, … , *t_m_* are finally extracted from *X*, PLS-R conducts the regression of *y_k_* for *t*_1_, *t*_2_, … , *t_m_*, and, then, *y_k_* is further expressed by a regression equation regarding the previous independent variables *x*_1_, *x*_2_, … , *x_p_*, *k* = 1, 2, … , *q*. The basic mathematical procedure of PLS-R is as follows.

(i) Suppose *X* = [*x*_1_, *x*_2_, … , *x_p_*]*_n_*_×_*_p_*, and *Y* = [*y*_1_, *y*_2_, … , *y_q_*]*_n_*_×_*_q_*. Standardizing *X* gives

2.1 $$x_{ij}^{*}=\frac{x_{ij}-\bar{x_{j}}}{s_{j}},$$

where i=1,2,…,n, j=1,2,…,p and $s_{j}={\left[\frac{1}{n}\sum_{\;j=1}^{n}{(x_{j}-\bar{x_{j}})}^{2}\right]}^{1/2}$.

Then, $E_{0}={\left[x_{1}^{*},x_{2}^{*},\ldots ,x_{j}^{*},\ldots ,x_{p}^{*}\right]}_{n\times p}$ is obtained.

*Y* is standardized in the same way, and, then, $F_{0}={\left[y_{1}^{*},y_{2}^{*},\ldots ,y_{j}^{*},\ldots ,y_{q}^{*}\right]}_{n\times q}$ is obtained.

(ii) Extract component:

2.2 $$w_{i}=\frac{E_{i-1}^{\prime }F_{i-1}}{∥E_{i-1}^{\prime }F_{i-1}∥},\;t_{i}=E_{i-1}w_{i},\;p_{i}=\frac{E_{i-1}^{\prime }t_{i}}{{∥t_{i}∥}^{2}}$$

and

2.3 $$E_{i}=E_{i-1}-t_{i}p_{i}^{\prime },$$

where *i* = 1, 2, 3, … , *m*, $m\leq \text{rank}(E_{0})$ and can be determined by cross-validation.

(iii) Solve regression coefficient vector:

2.4 $$r_{i}=\frac{F_{i-1}^{\prime }t_{i}}{{∥t_{i}∥}^{2}},\;i=1,2,3,\ldots ,m$$

and

2.5 $$w_{i}^{*}=\prod_{\;j=1}^{i-1}(I-w_{j}p_{j}^{\prime })w_{i}.$$

(iv) Obtain the PLS-R model:

2.6 $$\begin{array}{rl} & F_{0}=t_{1}r_{1}^{\prime }+t_{2}r_{2}^{\prime }+\ldots +t_{m}r_{m}^{\prime }+F_{m}=E_{0}w_{1}^{*}r_{1}^{\prime }+E_{0}w_{2}^{*}r_{2}^{\prime }+\ldots +E_{0}w_{m}^{*}r_{m}^{\prime }+F_{m}\\ & \;=E_{0}[\sum_{\;j=1}^{m}w_{j}^{*}r_{j}^{\prime }]+F_{m}=E_{0}B+F_{m} \end{array}$$

and

2.7 $$B=\sum_{\;j=1}^{m}w_{j}^{*}r_{j}^{\prime }\;,$$

where *B* is the regression coefficient vector of the PLS-R model, and *F_m_* is the residual matrix.

(v) Transfer the standardized variables into original variables to obtain the final PLS-R model.

The PLS-R method was applied to obtain the compressor map regression model. As PLS-R is a multiple linear regression method, we take the speed character and pressure character, which can always be easily measured as independent variables, and the flow character and efficiency character as dependent variables, and then organize the two independent variables into a group to introduce more variables. Its combination form is as follows:

2.8 $$z=\sum_{i=1}^{h}(\sum_{k=0}^{i}(a\cdot v_{1}^{i-k}\cdot v_{2}^{k}))+b,$$

where *z* is the flow character and efficiency character as dependent variables, *v*_1_ and *v*_2_ are the speed character and pressure character as independent variables, respectively, and *h* is the maximum fitting power of the polynomial.

Then:

2.9 $$i=1,\;k=0,\;x_{1}=v_{1}^{i-k}\cdot v_{2}^{k}=v_{1},$$

2.10 $$i=1,\;k=1,\;x_{2}=v_{1}^{i-k}\cdot v_{2}^{k}=v_{2},$$

2.11 $$i=2,\;k=0,\;x_{3}=v_{1}^{i-k}\cdot v_{2}^{k}=v_{1}^{2},$$

……

2.12 $$i=1,\;k=m,\;x_{{\;}_{[\sum_{i=1}^{l-1}\frac{(i+1)i}{2}+m+1]}}=v_{1}^{i-k}\cdot v_{2}^{k}=v_{1}^{l-m}\cdot v_{2}^{m},$$

…

2.13 $$\;\mathrm{and}\;\;\;\;\;\;\;\;\;\;i=h,\;k=h,\;x_{[\sum_{i=1}^{h}\frac{(i+1)i}{2}]}=v_{1}^{i-k}\cdot v_{2}^{k}=v_{2}^{h}.\;$$

Here

2.14 $$p=h+\sum_{i=1}^{h}\left(\sum_{k=0}^{i}\frac{(k+1)k}{2}\right)\;$$

and

2.15 $$y_{1}=z.$$

Then

2.16 $$\begin{array}{rl} X & ={\left[x_{1},x_{2},\ldots ,x_{p}\right]}_{n\times p,}\\ Y & ={\left[y_{1}\right]}_{n\times 1,} \end{array}\}\;$$

where *n* is the number of sample data points.

Then, the coefficient vector *a* and constant *b* can be derived by applying *X* and *Y* into the PLS-R algorithm. Finally, the PLS-R model of the compressor characteristics maps is established.

Compared with a traditional regression method, the main features of PLS-R are as follows [30].

(i) A strong correlation among independent variables is allowed during regression modelling.
(ii) It has a good tolerance to measurement noise.
(iii) Each regression coefficient for each independent variable can be interpreted in the PLS-R model.

To further match the nonlinearity of the compressor characteristics lines and to improve the prediction accuracy and shorten the calculation time, we chose the trigonometric function as the basis function of PLS(*ϕ*(*x*) = [1, sin *x*, cos *x*, sin 2*x*, cos 2*x*, … , sin *mx*, cos *mx*]^T^). This new prediction model is referred to as PLSN.

To compare the improvement of the prediction accuracy and computation time with the new model, the traditional power function polynomial-based PLS model (*ϕ*(*x*) = [1, *x*, *x*^2^, … , *x^b^*^−1^]^T^) is also listed in this paper, and is referred to as PLSO.

## 3. Application and analysis

**Figure 3.**
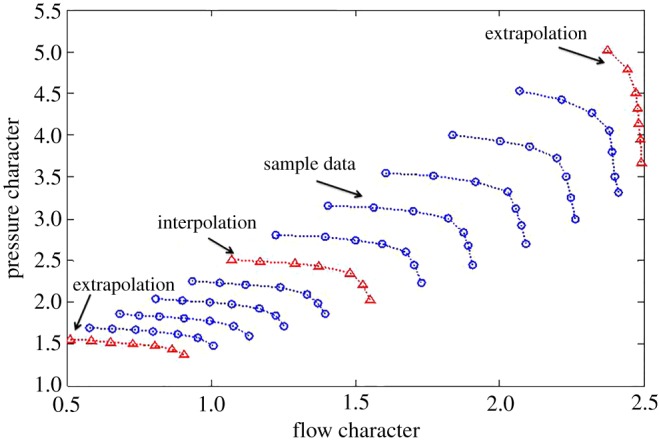
Flow characteristics map of a compressor.

**Figure 4.**
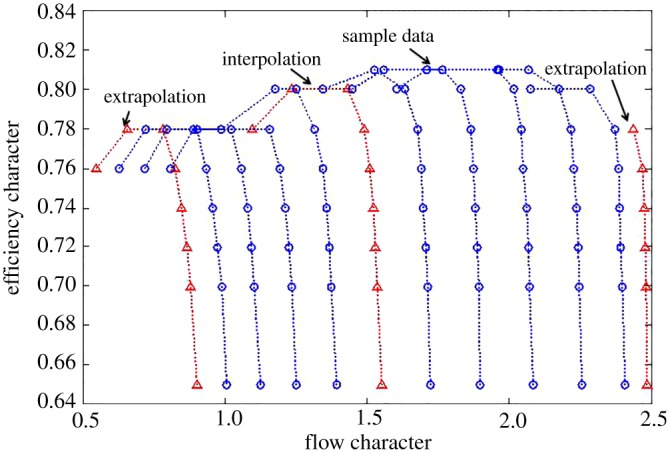
Efficiency characteristics map of a compressor.

Here, a certain compressor map obtained from a design manual of a centrifugal compressor, shown in figures 3 and 4 is used to test the effectiveness of the proposed method in which the sample data consist of nine speed lines. The speed lines of the test data are also shown in figures 3 and 4.

### 3.1. Flow characteristics map

**Figure 5.**
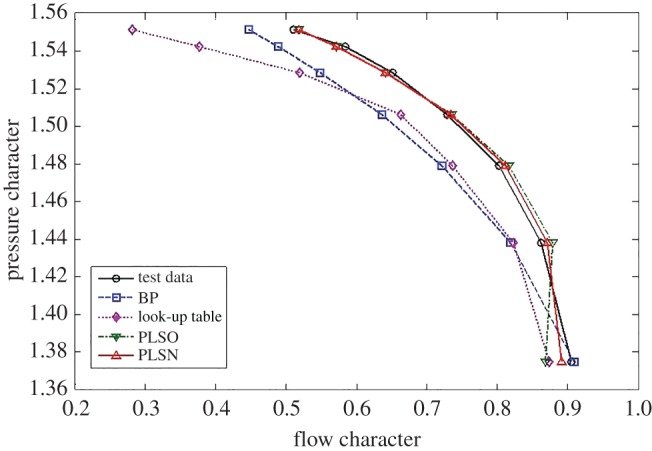
Extrapolation performance comparison at the lower part-load operating area.

**Figure 6.**
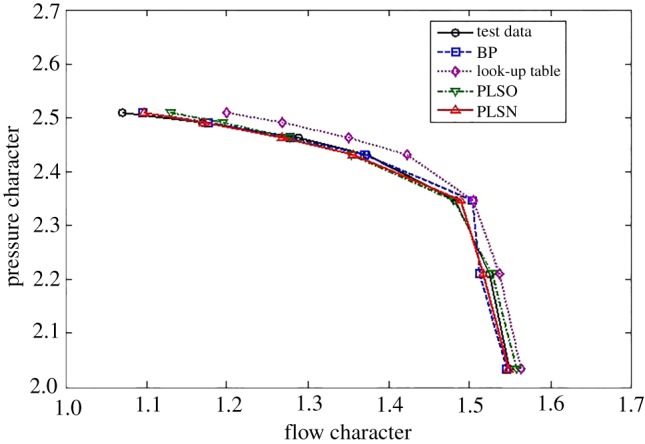
Interpolation performance comparison.

**Figure 7.**
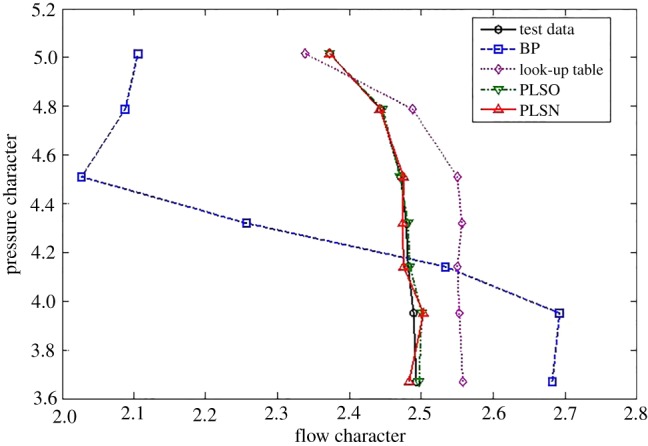
Extrapolation performance comparison at the upper part-load operating area.

To demonstrate the effectiveness of the proposed methods for the typical interpolated and extrapolated predictions, two other classical data-driven modelling methods, including the look-up table and feed-forward BPNN, are compared from the perspective of prediction accuracy and time consumption. For the flow characteristics map, the extrapolation and interpolation performance from the traditional methods and the proposed method are shown in figures 5–7.

A criterion is shown in equation (3.1) where the root mean square error (RMSE) of the difference between the predicted data and the test data is introduced to check the interpolation and extrapolation performance of the proposed method. The comparative results are shown in tables 2 and 3.

3.1 $$\text{RMSE}=\sqrt{\frac{\sum_{i=1}^{m}{\left[(z_{i,\mathrm{predicted}}-z_{i,\mathrm{test}})/z_{i,\mathrm{test}}\right]}^{2}}{m}},$$

where *z_i_*_,test_ is the *i*th test data, *z_i_*_,predicted_ is the corresponding prediction data of PLS-R model and *m* is the number of test data.

**Table 2.** Extrapolation and interpolation performance comparison of the traditional methods and the proposed method for the flow characteristics map.

algorithm	RMSE			
	EPL (%)	IPL (%)	EPU (%)	total (%)
look-up table	13.3236	6.9334	6.3699	9.4193
BP	7.6496	1.4385	27.3835	16.4362
PLSO	1.7510	2.6251	0.4814	1.8429
PLSN	0.9856	1.4937	0.7414	1.1184

**Table 3.** Time consumption comparison of the traditional methods and the proposed method for calculating one operating point.

algorithm	look-up table	BP	PLSO	PLSN
time consumption (s)	8 × 10^−4^	5.799 × 10^−3^	5.1 × 10^−5^	5 × 10^−6^

From figures 5–7 and table 2, the BPNN has a better interpolation performance than the other methods but also the worst extrapolation performance for the flow characteristics map, which is especially evident at the upper part-load operating area. In general, the proposed methods of PLSO and PLSN have an obvious improvement in both extrapolation and interpolation performance, especially for the extrapolation performance at the upper part-load operating area. From table 3, we can also see that the proposed method is very beneficial for engine real-time performance simulation and online monitoring. Furthermore, it is interesting to find that the PLS with the trigonometric function polynomial form as the basis function has an advantage over that with the polynomial form as the basis function, especially in computational time, which is reduced by an order of magnitude.

### 3.2. Efficiency characteristics map

**Figure 8.**
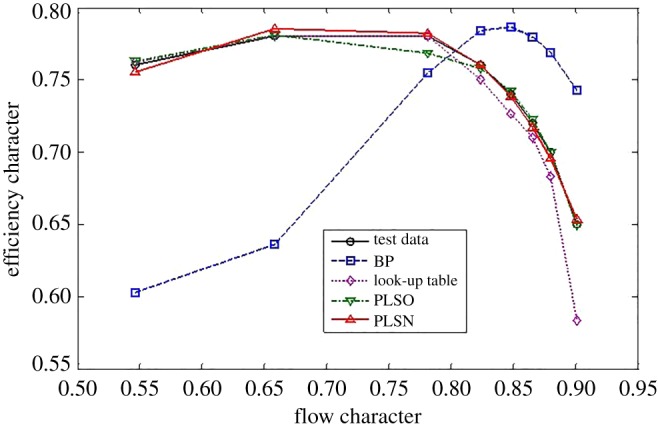
Extrapolation performance comparison at the lower part-load operating area.

**Figure 9.**
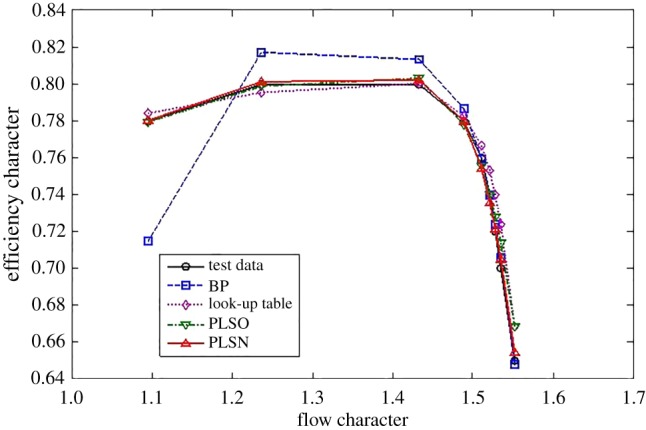
Interpolation performance comparison.

**Figure 10.**
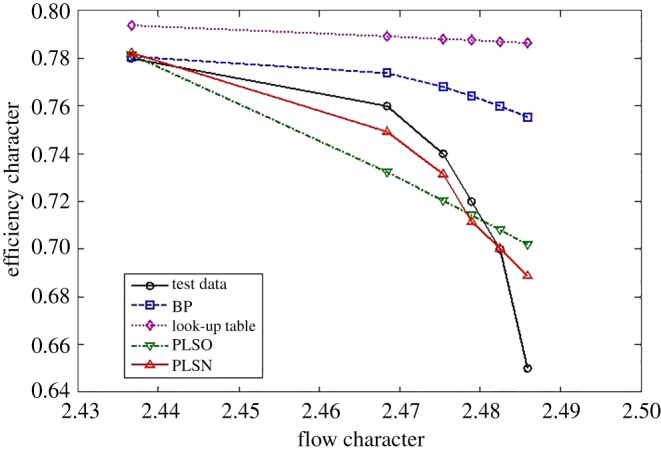
Extrapolation performance comparison at the upper part-load operating area.

For the efficiency characteristics map, the extrapolation and interpolation performance of the traditional methods and the proposed method are shown in figures 8–10.

The criterion shown in equation (3.1) is used to check the performance of the interpolation and extrapolation of the proposed method. The comparative results are shown in tables 4 and 5.

**Table 4.** Extrapolation and interpolation performance comparison of the traditional methods and the proposed method for the efficiency characteristics map.

algorithm	RMSE			
	EPL (%)	IPL (%)	EPU (%)	total (%)
look-up table	2.5276	1.3200	7.5256	4.2045
BP	9.0518	2.3180	5.4155	6.1849
PLSO	0.4384	0.8336	2.5670	1.4345
PLSN	0.3500	0.3430	1.7169	0.9261

**Table 5.** Time consumption comparison of the traditional methods and the proposed method for calculating one operating point.

algorithm	look-up table	BP	PLSO	PLSN
time consumption (s)	1.12 × 10^−4^	6.118 × 10^−3^	5.0 × 10^−5^	7 × 10^−6^

From figures 8–10 and table 4, the proposed methods of PLSO and PLSN have evident improvements in both the extrapolation and interpolation performance. For the interpolation performance, all four methods can predict the tendency of efficiency variations with the flow character. For the extrapolation performance, the BPNN lost the ability to predict the tendency of efficiency variations with the flow character in the lower part-load operating area, and only PLSN had the ability to predict the tendency of efficiency variations with the flow character in the upper part-load operating area. From table 5, we can also see that the proposed methods, especially PLSN, are very beneficial for engine real-time performance simulation and online monitoring because of their shorter computational time.

Furthermore, it is interesting to note that the PLS with the trigonometric function polynomial form as the basis function has several advantages over that with the power function polynomial form as the basis function. One is that the PLSN has a higher prediction accuracy than the PLSO despite the interpolation and extrapolation; the other is that the PLSN can predict the tendency of efficiency variation with the flow character successfully at the upper part-load operating area where PLSO had lost this ability; even more surprising is that the computational time of PLSN is much shorter than that of PLSO.

## 4. Conclusion and discussion

A multiple linear regression method, i.e. the PLS-R method, was proposed in this paper and applied to obtain the compressor map regression model based on known compressor characteristics maps. The following meaningful conclusions were determined.

(i) The proposed method has an evident improvement in both extrapolation and interpolation performance.
(ii) PLS with the trigonometric function polynomial form as the basis function has the advantage of both expressive precision and time consumption over that with the power function polynomial form as the basis function.
(iii) Compared with [25,26], the new PLS has a similar or even slightly improved prediction accuracy. It also avoids the complex structure of the ANN-PLS and complicated kernel function of KPLS.
(iv) The PLS-R model may improve the real-time calculating performance during the dynamic performance simulation of a diesel engine performance model.
(v) It can be expected that the application of PLS-R modelling has a certain reference value to improve the accuracy for thermodynamic performance modelling of a diesel engine.

## Data accessibility

Our primary data can be found at the website: http://www.jtp.com.cn/cpzx/zyq/jthxl/223820.htm.
